# Leaf and Root Extracts from *Campomanesia adamantium* (Myrtaceae) Promote Apoptotic Death of Leukemic Cells via Activation of Intracellular Calcium and Caspase-3

**DOI:** 10.3389/fphar.2017.00466

**Published:** 2017-08-14

**Authors:** Jaqueline F. Campos, Priscilla P. de Toledo Espindola, Heron F. V. Torquato, Wagner D. Vital, Giselle Z. Justo, Denise B. Silva, Carlos A. Carollo, Kely de Picoli Souza, Edgar J. Paredes-Gamero, Edson L. dos Santos

**Affiliations:** ^1^Research Group on Biotechnology and Bioprospecting Applied to Metabolism, Federal University of Grande Dourados Dourados, Brazil; ^2^Department of Biochemistry, Federal University of São Paulo São Paulo, Brazil; ^3^Faculty of Pharmacy, Braz Cubas University Mogi das Cruzes, Brazil; ^4^Interdisciplinary Center of Biochemistry Investigation, University of Mogi das Cruzes Mogi das Cruzes, Brazil; ^5^Department of Pharmaceutical Sciences, Federal University of São Paulo São Paulo, Brazil; ^6^Laboratory of Natural Products and Mass Spectrometry, Federal University of Mato Grosso do Sul Mato Grosso do Sul, Brazil

**Keywords:** natural products, medicinal plant, LC-MS, cancer, bioprospecting

## Abstract

Phytochemical studies are seeking new alternatives to prevent or treat cancer, including different types of leukemias. *Campomanesia adamantium*, commonly known as guavira or guabiroba, exhibits pharmacological properties including antioxidant, antimicrobial, and antiproliferative activities. Considering the anticancer potential of this plant species, the aim of this study was to evaluate the antileukemic activity and the chemical composition of aqueous extracts from the leaves (AECL) and roots (AECR) of *C. adamantium* and their possible mechanisms of action. The extracts were analyzed by LC-DAD-MS, and their constituents were identified based on the UV, MS, and MS/MS data. The AECL and AECR showed different chemical compositions, which were identified as main compounds glycosylated flavonols from AECL and ellagic acid and their derivatives from AECR. The cytotoxicity promoted by these extracts were evaluated using human peripheral blood mononuclear cells and Jurkat leukemic cell line. The cell death profile was evaluated using annexin-V-FITC and propidium iodide labeling. Changes in the mitochondrial membrane potential, the activity of caspases, and intracellular calcium levels were assessed. The cell cycle profile was evaluated using propidium iodide. Both extracts caused concentration-dependent cytotoxicity only in Jurkat cells via late apoptosis. This activity was associated with loss of the mitochondrial membrane potential, activation of caspases-9 and -3, changes in intracellular calcium levels, and cell cycle arrest in S-phase. Therefore, the antileukemic activity of the AECL and AECR is mediated by mitochondrial dysfunction and intracellular messengers, which activate the intrinsic apoptotic pathway. Hence, aqueous extracts of the leaves and roots of *C. adamantium* show therapeutic potential for use in the prevention and treatment of diseases associated the proliferation of tumor cell.

## Introduction

Leukemias are cancers that affect white blood cells (Yan et al., 2014), which are responsible for activation of the immune system. Leukemia can be classified as lymphocytic or myeloid according to the cellular origin and as either acute or chronic according to the progression of the disease.

To control or inhibit the proliferation of altered leukemic cells in patients, various treatment strategies can be combined, including chemical treatment, radiological treatment, and stem cell transplantation. In this context, the natural products have been suggesting novel alternatives to prevent or treat cancer, including different types of leukemias (Asmaa et al., 2014; Kinghorn, 2015).

*Campomanesia adamantium* (Cambess.) O. Berg (Myrtaceae), commonly known as guavira or guabiroba, is a small tree endemic to Brazil that produces edible fruits that are widely used in the production of liqueurs, juices, and jellies (Coutinho et al., 2008; Pavan et al., 2009). The leaves and fruits of *C. adamantium* are used in the traditional medicine as anti-inflammatory, antidiarrheal, and are effective against urinary infections diseases (Vieira et al., 2011), and the roots are used in the treatment of diabetes (Coutinho et al., 2008).

Studies on the chemical composition from *C. adamantium* have indicated the presence of phenolic compounds, such as chalcones (Pavan et al., 2009; Pascoal et al., 2011, 2014), flavanones and flavonols (Coutinho et al., 2010), as well as gallic acid, and ellagic acid (Espindola et al., 2016).

Besides, previous studies have reported antimicrobial (Pavan et al., 2009; Cardoso et al., 2010), antiproliferative (Pascoal et al., 2014), anti-inflammatory, antidepressant, antihyperalgesic, and antidiarrheal activities of the fruits (Lescano et al., 2016; Souza et al., 2017). The roots exhibits antioxidant and antihyperlipidemic effects (Espindola et al., 2016). In adition, the essential oil of the leaves exhibits antimicrobial and antioxidant properties (Coutinho et al., 2009), and leaf extracts show anti-inflammatory, antinociceptive (Ferreira et al., 2013), antioxidant (Coutinho et al., 2010; Pascoal et al., 2011), and antiproliferative activity against prostate cancer cells by decreased the expression of NFkB1 and induction of apoptosis (Pascoal et al., 2014). However, to the best of our knowledge, no previous studies have described the activities of this plant species in leukemic cells.

Considering the anticancer potential of *C. adamantium*, the aim of this study was to evaluate the antileukemic activity of aqueous extracts of its leaves and roots, their mechanisms of action as well as its chemical composition.

## Materials and methods

### Botanical specimens and preparation of extracts

*C. adamantium* O. Berg leaves and roots were collected following the identification of the plant and authorization of the SISBIO (*Sistema de Autorizaç*ão e Informação em Biodiversidade, permit number 54470).

Leaves and roots of *C. adamantium* O. Berg were collected in Dourados, in the state of Mato Grosso do Sul, Brazil (coordinates: 22° 02′ 47.9 S′ and 055° 08′ 14.3′ W). The samples were cleaned, dried in a convection oven at 45°C, and ground using a Croton knife mill. An exsiccated sample was deposited in the Herbarium of the Federal University of Grande Dourados, Mato Grosso do Sul, Brazil, with registration number 4108.

Extracts were prepared from the powdered material using an accelerated solvent extractor (ASE®-150, Dionex), as described by Espindola et al. (2016). The samples were placed in a cell of 100 mL and extracted with distilled water at temperature of 125°C through two 5 min static cycles, with an 80% flush volume and a 60 s purge. The extracts were lyophilized to obtain the dry extract. Thus, the aqueous extracts of *Campomanesia* leaves (AECL) and roots (AECR) were obtained with a yield of 13 and 6%, respectively.

### Identification of constituents by liquid chromatography coupled to array diode detector and mass spectrometry (LC-DAD-MS)

The analyses were performed at a Shimadzu Prominence UFLC coupled to a diode array detector (DAD) and a mass spectrometer (MicrOTOF-Q III, Bruker Daltonics) with an electrospray ion source. Kinetex C18 (2.6 μm, 150 × 2.1 mm, Phenomenex) chromatographic column was used. The AECL and AECR extracts were prepared at concentration 1 mg/mL and the volume of 1 μL was injected on the chromatography. The flow rate and oven temperature were 0.3 mL/min and 50°C, respectively. Formic acid 0.1% (v/v) in both water (solvent A) and acetonitrile (solvent B) were used as mobile phase, applying the following gradient elution profile: 0–8 min at 3% B, 8–30 min at 3–25% B, 30–60 min at 25–80% B, and 60–63 min at 80% B. The MS analyses were carried out both ion modes (positive and negative), applying 2.5 kV of capillary voltage. Nitrogen was used as nebulizer gas (4 Bar), dry (9 L/min) and collisional gas.

### Cell line and culture conditions

Human peripheral blood mononuclear cells from healthy donors were collected after informed patient consent. Separation of mononuclear cells was performed by gradient centrifugation methods using Ficoll Histopaque-1077 (1.077 g/cm^3^) (Sigma–Aldrich, Germany) follow the manufacturer's instructions at 400 g for 30 min. The use of human samples was approved by the local Ethical Committee of the University Center of the Grande Dourados under protocol number 123/12.

The Jurkat (human acute T lymphocytic leukemia) cell line was cultured in suspension in RPMI 1640 medium (Cultilab, Brazil) supplemented with 10% fetal calf serum (FBS, Cultilab, Brazil), 100 U/mL penicillin, and 100 μg/mL streptomycin in a humidified atmosphere at 37°C under 5% CO_2_.

### Cytotoxic activity

Peripheral blood mononuclear cells and Jurkat cells were seeded (10^5^ cells/mL) in 96-well microplates containing medium with vinblastine (positive control), EACL or EACR at different concentrations. After incubation for 24 h, the treatments were removed and 100 μL of 10% Alamar Blue solution was added. After 5 h, the fluorescence was read at 530 nm (Ex) and 590 nm (Em) in a microplate reader FlexStation 3 (Molecular Devices, USA). Each experiment was performed in triplicate.

### Cell death profile

The cell death profile was evaluated according to the methods described by Paredes-Gamero et al. (2012), with minor modifications. Jurkat cells were seeded in 96-well plates (10^5^ cells/mL) and cultured in medium containing 10% FBS for 24 h with IC_50_ of AECL (40 μg/mL) or AECR (80 μg/mL) and the double of these concentrations. After this period, the cells were washed with PBS and resuspended in annexin labeling buffer (0.01 M Hepes, pH 7.4, 0.14 M NaCl, and 2.5 mM CaCl_2_). The suspensions were labeled with annexin-FITC and propidium iodide (PI) (Becton Dickinson, USA) according to the manufacturer's instructions, and the cells were incubated at room temperature for 15 min. Finally, 10,000 events were collected per sample through analysis in a Accuri C6 flow cytometer (Becton Dickinson, USA) using FlowJo v10.2 LCC software (Oregon, USA).

### Measurement of the mitochondrial membrane potential (ΔΨmit)

Mitochondrial depolarization was evaluated through the incorporation of JC-1 (5,5′,6,6′–tetrachloro—1,1′,3,3′—tetraethylbenzimidazolylcarbocyanine iodide; Molecular Probes, Eugene, OR, USA) according to the method described by Moraes et al. (2013). JC-1 is cationic dye that shows potential-dependent accumulation in mitochondria via fluorescence emission. Cells that stained red were classified as exhibiting a high mitochondrial membrane potential, while those that stained green were classified as exhibiting a low membrane potential. For these assays, cells were seeded in 24-well plates (10^5^ cells/mL), where they were cultured in medium containing 10% FBS and treated with IC_50_ of the AECL or AECR, for 24 h. After treatment, the cells were centrifuged and incubated with JC-1 (1 μg/mL) at room temperature for 15 min. Fluorescence was analyzed in a FACScalibur Flow Cytometer (Becton Dickinson, USA) using CellQuest software (10,000 events were collected per sample).

### Western blot analysis

Antibodies against actin, Bcl-2 and Bax were obtained from Santa Cruz Biotecnology (Santa Cruz, CA, USA), whereas the monoclonals anti-cleaved caspase-9 and anti-cleaved caspase-8 antibodies were from Cell Signaling Technologies (Beverly, MA, USA). Horseradish peroxidase-conjugated secondary antibodies against rabbit (goat anti-rabbit), mouse (goat anti-mouse), and goat (donkey anti-goat) were from Millipore (Temecula, CA, USA).

After treatment with IC_50_ of the AECL or AECR, cells were harvested in a lysis buffer (50 mM Tris-HCl, pH 7.4), 1% Tween-20, 0.25% sodium deoxycholate, 150 mM NaCl, 1mM EGTA, 1mM o-Vanadate, 1mM NaF, and 1X protease inhibitor cocktail (Thermo Fisher Scientific, Rockford, IL, USA) on ice for 2 h, and after clarification, protein concentration was determined using the Pierce BCA Protein Assay kit (Thermo Fisher Scientific). Equal volume of 2X SDS gel loading buffer was added and samples were boiled for 5 min. Cellular extracts were subjected to SDS-PAGE (10%) and transferred to nitrocellulose membranes. Blots were probed overnight at 4°C with appropriate primary antibodies at 1:1,000 dilutions. After washing in TBS/0.05% Tween-20, membranes were incubated with anti-goat, anti-rabbit, or anti-mouse horseradish peroxidase-conjugated secondary antibody at 1:3,000 dilutions for 1 h. Blots were imaged using Pierce SuperSignal West Pico Chemiluminescent Substrate (Thermo Fisher Scientific) on a LAS-3000 imaging system.

### Caspase-3 activity

The activation of caspase-3 was evaluated via flow cytometry according to the method described by Moraes et al. (2013), with minor modifications. Cells were seeded in 24-well plates (10^5^ cells/mL), where they were cultured in medium containing 10% FBS and treated with IC_50_ of the AECL or AECR, for 24 h. After treatment, the cells were centrifuged, washed, and fixed in 2% paraformaldehyde in PBS for 30 min. The cells were then washed with glicin (0.1 M) in PBS, permeabilized with 0.01% saponin for 30 min, and blocked in PBS containing 1% BSA for 30 min at room temperature. Subsequently, the cells were incubated with an anti-active-caspase-3 monoclonal antibody conjugated with FITC (BD-Pharmingen, San Diego, CA, USA) in the dark at room temperature for 40 min. After incubation, fluorescence was analyzed in a FACScalibur Flow Cytometer (Becton Dickinson, USA) using CellQuest software (10,000 events were collected per sample).

### Pan-caspase inhibitor and intracellular calcium

The importance of caspases and calcium for AECL or AECR cytotoxicity was evaluated according to the method described by Bechara et al. (2014), with minor modifications. Briefly, Jurkat cells that had been pretreated for 1 h with 10 μM of the Z-VAD-FMK (general caspases inhibitor) or 10 μM of the BAPTA-AM (a calcium chelator) before AECL or AECR incubation for 24 h, both with IC_50_ values, were subjected to trypan blue exclusion assays. To assess cell viability, control and treated cells were resuspended in equal volumes of medium and trypan blue (0.05% solution) and were counted using a hemocytometer chamber.

### Ca^2+^ measurement

Cytosolic Ca^2+^ concentration [Ca^2+^]_cyt_ was determined by incubating 10^5^ Jurkat cells/well in black 96-well microplates with the Fluo-4 Direct Calcium Assay reagent according to the manufacturer's instructions (Life, USA). After 1 h of indicator incorporation at 37°C, cells were stimulated with IC_50_ of the AECL or AECR and the fluorescence was quantified in a FlexStation 3 microplate reader (Molecular Devices, USA). Ionomycin 0.1 μM, was used as positive control to obtain the maximal fluorescence. The Fluo-4 was excited at 490 nm, and the emission was detected at 525 nm (Leon et al., 2011). The records of fluorescence intensity as a function of time correspond to the mean of experiment performed in quadruplicate.

### Cell cycle phases

To determine the cell cycle distribution, Jurkat cells were seeded (10^5^ cells/mL) and cultured in medium containing 10% FBS and were treated with IC_50_ of the AECL or AECR, for 24 h. After this period, the cells were washed, fixed and permeabilized as described previously. Then, the cells were treated with 4 μg/mL RNase type I for 1 h at 37°C and resuspended in PBS. The cells were subsequently stained with 5 μg/mL PI and analyzed (12,000 events collected per sample) in an Accuri C6 flow cytometer (Becton Dickinson, USA). The DNA content was evaluated using an FL2A detector on a linear scale. The dead cells were excluded to cell cycle analysis. The analyses of the cell percentage in the G1/S/G2 phases were performed using FlowJo v10.2 LCC software.

### Intracellular protein labeling

To investigate the mechanisms of extracts-induced cell cycle arrest, Jurkat cells were seeded in 24-well plates (10^5^ cells/mL), where they were cultured in medium containing 10% FBS and treated with IC_50_ of the AECL or AECR, for 24 h. After this period, were fixed with 2% paraformaldehyde for 10 min, washed with BD Perm/Wash buffer and permeabilized with BD Perm Buffer III for 30 min. Then, the cells were labeled with 5 μL Ki-67-FITC antibody (Becton Dickinson, USA). To label phosphorylated retinoblastoma protein (p-Rb) anti-p-Rb (Ser780) (Cell Signaling, USA) antibody was used. After permeabilization intracellular protein was incubated for 1 h. Then, anti-rabbit IgG secondary antibody conjugated with Alexa Fluor 488 (Life, USA) were used for 40 min. Protein analyses were performed by quantification of the fluorescence geometric mean (Gm). The cells were analyzed in an Accuri C6 flow cytometer (Becton Dickinson, USA) using FlowJo v10.2 LCC software (Torquato et al., 2017).

### Statistical analyses

The data are shown as the mean ± standard error of the mean (SEM) and were analyzed for statistically significant differences between groups. Student's *t*-test was employed for comparisons between two groups, and one-way analysis of variance (ANOVA) followed by Dunnett's test was employed for comparisons of more than two groups using Prism 5 GraphPad Software. The results were considered significant when *P* < 0.05.

## Results

### Identification of constituents from the extracts

**Figure 1:**
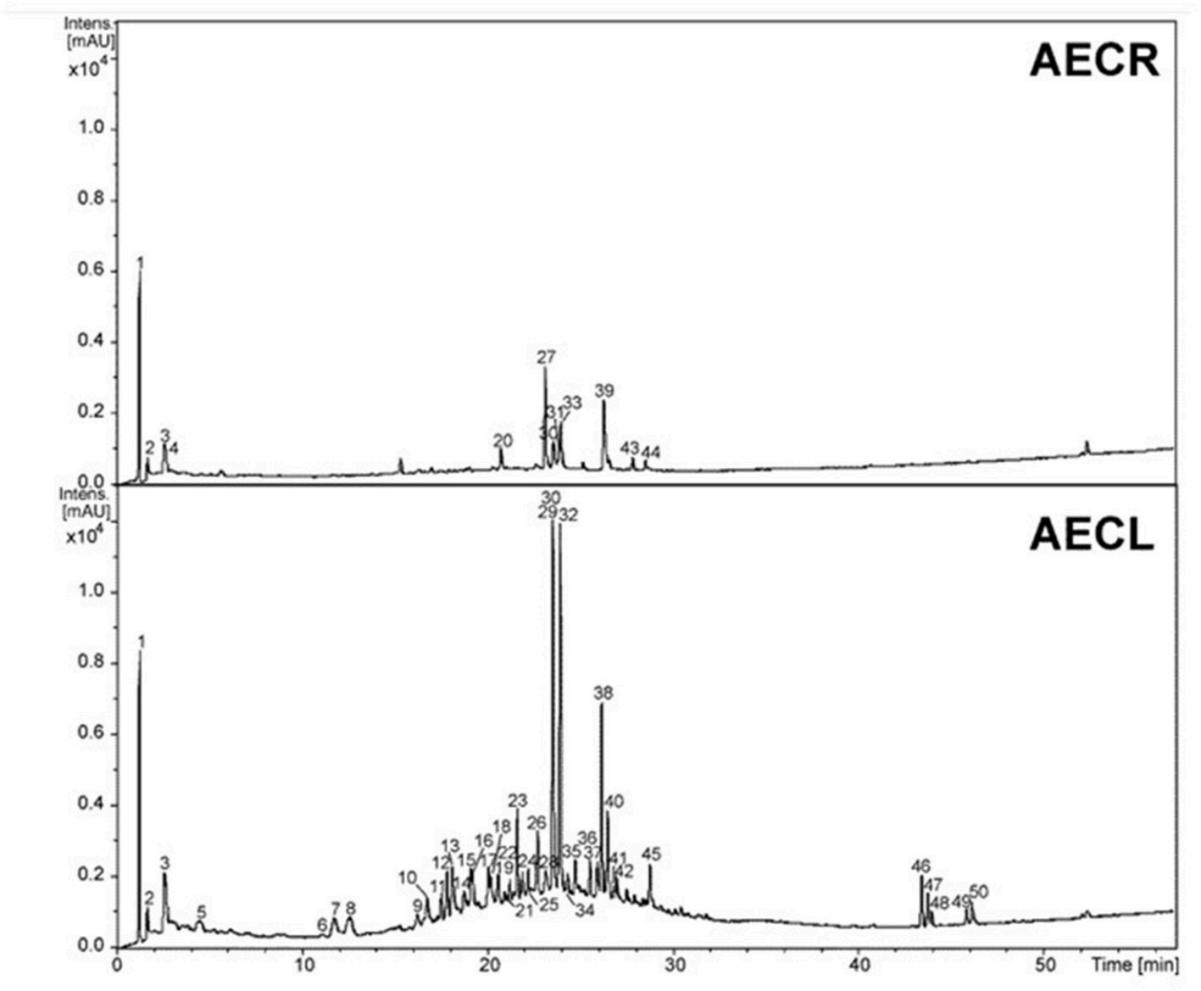
Chromatographic profile at wavelength 230–350 nm of the aqueous extracts of roots (AECR) and leaves (AECL) from *C. adamantium*.

**Figure 2:**
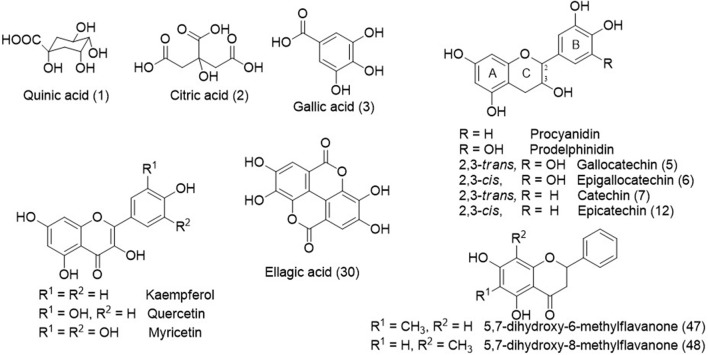
Chemical structures some identified compounds and aglycones from *C. adamantium*.

The constituents from AECR and AECL were identified based on the UV, high resolution MS and MS/MS data (Figure 1, Table 1) and the comparison to the published data (Fabre et al., 2001; March et al., 2006; Ferreres et al., 2013; Guaratini et al., 2014; Zhang et al., 2016). The main compounds identified from AECR were di-hexoside/quinic acid (**1**), ellagic acid *O*-pentoside (**27**), ellagic acid (**30**), *O*-methyl ellagic acid *O*-hexoside (**31**), ellagic acid *O*-deoxyhexoside (**33**), and *O*-methyl ellagic acid sulfate (**39**) (Table 1). In addition, other constituents were identified such as gallic acid (**3**), ellagic acid *O*-hexoside (**20**), *O*-methyl ellagic acid *O*-deoxyhexoside (**43**), and *O*-dimethyl ellagic acid sulfate (**44**) (Figure 2).

**Table 1:** Identification of the constituents of aqueous extract from *C. adamantium* by LC-DAD-MS.

**Peak**	**Retention time (min)**	**Compound**	**UV (nm)**	**Molecular formula**	**Negative mode (*****m/z*****)**		**Positive mode (*****m/z*****)**	
					MS [M-H]^–^ (^*^)	**MS/MS**	MS (^*^)	**MS/MS**
1	1.1	Di-hexoside	–	C_12_H_22_O_11_	341.1084 (1.5)	–	–	–
		Quinic acid	–	C_7_H_12_O_6_	191.0545 (8.3)	–	193.0700 (0.7)	–
2	1.7	Citric acid	–	C_6_H_8_O_7_	191.0193 (2.2)	–	193.0350 (0.7)	–
3	2.6	Gallic acid^st-1^	270	C_7_H_6_O_5_	169.0141 (1.1)	–	171.0274 (7.9)	–
4	2.6	NI	270	C_10_H_18_O_8_	265.0931 (0.9)	–	289.0891 (3.5)^Na^	–
5	4.6	Gallocatechin^st-2^	275	C_15_H_14_O_7_	305.0654 (4.1)	219, 179, 165	307.0815 (0.9)	195, 163, 159
6	11.2	Epigallocatechin^st-3^	278	C_15_H_14_O_7_	305.0664 (1.0)	–	307.0814 (0.5)	–
7	11.7	Catechin^st-4^	275	C_15_H_14_O_6_	289.0711 (2.4)	–	291.0851 (4.0)	189, 161, 147
8	12.5	PCY-PCY dimer (B type)	278	C_30_H_26_O_12_	577.1350 (0.3)	407, 289, 245, 161	579.1497 (0)	409, 287, 271, 257, 247, 233, 191, 163
9	16.2	PCY-PCY dimer (B type)	278	C_30_H_26_O_12_	577.1348 (0.6)	289	579.1515 (3.1)	409, 287, 271, 257, 233, 163
		PCY-PCY-PCY trimer (B type)		C_45_H_39_O_18_	865.1980 (0.6)	577, 289	867.2102 (3.3)	–
10	16.7	NI	256, 275	C_13_H_18_O_5_	253.1078 (1.4)	195	255.1213 (5.6)	–
11	17.4	PCY-PCY dimer (B type)	280	C_30_H_26_O_12_	577.1331 (2.0)	407, 339, 289, 245, 161	579.1497 (0)	409, 287, 271, 233, 163
12	17.8	Epicatechin^st-5^	278	C_15_H_14_O_6_	289.0711 (2.2)	245, 179	291.0863 (0.1)	189, 161
13	18.1	Hydrolysable tannin	276	C_48_H_32_O_32_	1119.0782 (2.0)	1057, 933, 913, 887, 425, 299, 273	1143.0767 (0.1)^Na^	951, 471, 453, 427, 337, 261
14	18.8	PCY-PDE O- gallate (B type)	276	C_37_H_30_O_17_	745.1381 (3.9)	407, 305, 249	747.1565 (0.9)	425, 407, 287, 275, 247
15	19.1	NI	275, 350	C_20_H_18_O_13_	465.0660 (3.1)	332, 287	467,0863 (3.0)	335, 189, 169
16	19.2	NI	278, 360	C_21_H_20_O_13_	479.0817 (2.9)	332, 287	481.0985 (1.8)	335
17	20.1	Hydrolysable tannin	276	C_41_H_28_O_27_	951.0705 (4.2)	933, 301, 273	975.0703 (0.7)^Na^	-
18	20.2	PCY-PCY *O*-gallate (B type)	280	C_37_H_30_O_16_	729.1435 (3.6)	407, 289, 169	731.1629 (3.1)	409, 283, 271, 259, 153
19	20.6	PCY-PCY-PCY (B type)	280	C_45_H_38_O_18_	865.1981 (0.5)	407, 287	867.2134 (0.4)	427, 409, 291, 289, 275, 247
		Myricetin *O*-pentoside	–, 360	C_20_H_18_O_12_	449.0720 (1.2)	316	451.0893 (4.8)	-
20	20.7	Ellagic acid *O*-hexoside	252, 360	C_20_H_16_O_13_	463.0491 (5.8)	301	465.0692 (5.8)	303, 275, 257, 247, 229
21	21.1	PCY-PCY dimer (B type)	280	C_30_H_26_O_12_	577.1341 (1.8)	407, 289	579.1500 (0.3)	409, 287, 271, 257, 163
22	21.2	PCY-PCY *O*-gallate (B type)	280	C_37_H_30_O_16_	729.1432 (3.9)	451, 407, 289, 271, 169	731.1597 (1.4)	409, 287, 275, 247, 163, 151
23	21.7	Myricetin *O*-pentoside	275, 350	C_20_H_18_O_12_	449.0703 (5.1)	316, 287, 271	451.0876 (1.1)	319, 273, 245
24	21.9	Myricetin *O*-hexoside	266, 360	C_21_H_20_O_13_	479.0833 (0.49)	316	481.0975 (0.2)	319
25	22.2	Myricetin *O*-hexoside	265, 355	C_21_H_20_O_13_	479.0808 (4.9)	316	481.0965 (2.4)	319
26	22.7	Myricetin *O*-pentoside	270, 355	C_20_H_18_O_12_	449.0711 (3.3)	316, 287, 271	451.0858 (3.0)	319, 273, 245
27	23.1	Ellagic acid *O*-pentoside	253, 360	C_19_H_14_O_12_	433.0425 (2.8)	301	435.0571 (3.0)	303, 275, 257
28	23.3	PCY *O*-gallate	280	C_22_H_18_O_10_	441.0807 (4.6)	169	433.0960 (2.9)	–
29	23.6	Myricetin *O*-pentoside	268, 355	C_20_H_18_O_12_	449.0711 (3.3)	316, 287, 271	451.0858 (3.0)	319, 273, 245
30	23.6	Ellagic acid^st-6^	252, 365	C_14_H_6_O_8_	301.0003 (4.5)	-	303.0128 (2.3)	275, 257, 247, 201
31	23.9	*O*-Methyl ellagic acid *O*-hexoside	250, 360	C_21_H_18_O_13_	477.0664 (2.1)	315	479.0798 (4.7)	317, 302, 285, 257
32	24.0	Myricetin *O*-deoxyhexoside	260, 350	C_21_H_20_O_12_	463.0857 (5.4)	316, 287, 271	465.1035 (0.8)	319, 273, 245, 165, 153
33	24.0	Ellagic acid *O*-deoxyhexoside	253, 360	C_20_H_16_O_12_	447.0562 (1.7)	300	449.0723 (1.9)	303, 285, 257
34	24.3	Quercetin *O*-hexoside	–, 354	C_21_H_20_O_12_	463.0865 (3.6)	300	465.1031 (0.7)	303
35	24.8	Quercetin *O*-hexoside	268, 354	C_21_H_20_O_12_	463.0872 (2.1)	300	465.1017 (2.3)	303
36	25.5	Quercetin *O*-pentoside	260,355	C_20_H_18_O_11_	433.0769 (1.6)	300, 271	435.0911 (2.5)	303
37	25.9	Quercetin *O*-pentoside	265,352	C_20_H_18_O_11_	433.0757 (4.5)	300, 271	435.0925 (0.8)	303
38	26.2	Quercetin *O*-pentoside	258,352	C_20_H_18_O_11_	433.0757 (4.6)	300, 271, 255, 243, 179	435.0922 (0.2)	303, 257, 229, 165, 153
39	26.3	*O*-Methyl ellagic acid sulfate	247, 360	C_15_H_8_O_11_S	394.9712 (0.7)	315, 300	396.9844 (3.9)	317, 302, 285, 257
40	26.5	Myricetin *O*-(*O*-galloyl)-pentoside	268, 355	C_27_H_22_O_16_	601.0796 (6.5)	449, 316, 299, 283	603.1002 (3.5)	319, 285, 153
41	26.9	Kaempferol *O*-hexoside	265, 350	C_21_H_20_O_11_	447.0902 (6.9)	284	449.1071 (1.1)	287
42	27.0	Quercetin *O*-deoxyhexoside	268,350	C_21_H_20_O_11_	447.0911 (4.8)	300, 271	449.1082 (0.8)	303
43	27.8	*O*-Methyl ellagic acid *O*-deoxyhexoside	248, 365	C_21_H_18_O_12_	461.0737 (2.6)	317, 302, 285, 257	463.0892 (4.6)	315, 300
44	28.5	O-Dimethyl ellagic acid sulfate	265, 360	C_16_H_10_O_11_S	408.9868 (0.8)	329, 314, 299	411.0026 (2.4)	–
45	28.8	Quercetin *O*-(*O*-galloyl)-pentoside	268, 355	C_27_H_22_O_15_	585.0886 (1.6)	433, 301, 300, 283, 169	587.1023 (1.4)	303, 285, 171, 153
46	43.4	NI	290, 335^sh^	C_13_H_18_O_4_	237.1130 (1.0)	193, 175, 167	239.1268 (4.3)	206, 191, 178, 163
47	43.7	5,7-dihydroxy-6-methylflavanone	292, 335^sh^	C_16_H_14_O_4_	269.0820 (0.3)	227, 199, 183, 171, 165	271.0956 (3.3)	167
48	43.9	5,7-dihydroxy-8-methylflavanone	292, 337^sh^	C_16_H_14_O_4_	269.0810 (3.3)	227, 165	271.0967 (0.8)	167
49	45.8	NI	335	C_14_H_20_O_4_	251.1288 (0.4)	233, 207, 193, 167	253.1434 (0.2)	205, 165, 152
50	46.1	NI	335	C_14_H_20_O_4_	251.1279 (4.0)	233, 207, 189, 167	253.1436 (0.5)	235, 205, 191, 177

* *, error in ppm;^Na^, [M+Na]^+^;^sh^ shoulder; PCY, procyanidin; PDE, prodelphinidin NI, non-identified;^st^, confirmed by authentic standard (1- MS^2^ neg (m/z): 125; 2- MS^2^ neg (m/z): 219, 179, 165; 3- MS^2^ neg (m/z): 287, 261, 179; 4- MS^2^ pos (m/z): 207, 189, 161; 5- MS^2^ pos (m/z): 189, 161, 6- MS^2^ pos (m/z): 275, 257, 247)*.

While from AECL, forty-two compounds have been identified. The main constituents identified were di-hexoside/quinic acid (**1**), myricetin *O*-pentoside (**23, 26, 29**), myricetin *O*-deoxyhexoside (**32**), quercetin *O*-pentoside (**38**), and myricetin *O*-(*O*-galloyl)-pentoside (**40**).

The peaks **5–9, 11–12, 14, 18–19**, **21–22**, and **28** (Table 1) presented a similar UV (λ_max_ ≈ 280 nm), which are compatible to flavan-3-ols (Markham, 1982). The typical fragmentation for condensed tannins was observed for these chromatographic peaks, including the heterocyclic ring fission, reto-Diels-Alder (RDA) and quinone methide reactions (Gu et al., 2003; Guaratini et al., 2014; Nocchi et al., 2017). The condensed tannins esterified with gallic acid were confirmed by fragment ion at *m/z* 169 [M-H-flavan-3-ol units]^−^, such as for **14, 18**, **22**, and **28**. The monomers were confirmed by co-injection of authentic standard as gallocatechin (**5**), epigallocatechin (**6**), catechin (**7**), and epicatechin (**12**)(Figure 2).

The compounds **19, 23–26, 29, 32, 34–38**, **40–42**, and **45** exhibited UV spectra relative to flavonols (Markham, 1982). The losses of 162, 146, 132, and 152 *u* confirmed the presence of hexoside, deoxyhexoside, pentoside, and gallic acid, respectively. The identification of aglycones was based on product ions yielded from C-ring fissions, as widely described in the literature (Fabre et al., 2001; March et al., 2006), and they corroborated to the aglycones quercetin, kaempferol, and myricetin. In addition, the compounds **47** and **48** showed intense ions at *m/z* 269 [M-H]^−^ and 271 [M+H]^+^, compatible to molecular formula C_16_H_14_O_4_ and UV spectra characteristic of flavanone. The determination of the methyl position in A-ring was established from elution time described for 5,7-dihydroxy-6-methylflavanone and 5,7-dihydroxy-8-methylflavanone, which were isolated from *C. adamantium* (Coutinho et al., 2010).

The compounds **20, 27, 30, 31, 33**, **39, 43**, and **44** showed UV spectra compatible to ellagic acid (Ferreres et al., 2013). From *m/z* 303.0128 [M+H]^+^ (C_14_H_7_O_8_^+^, error 4.5 ppm) of compound **30** were yielded the product ions at *m/z* 275, 257, and 247, which are relative to subsequent eliminations of CO (28 *u*) and water molecules and they were compatible to ellagic acid (confirmed by co-injection of the standard) (Ferreres et al., 2013; Zhang et al., 2016). The losses of 15 and 80 *u* confirmed the presence of methoxyl (**31, 39, 44**) and sulfate substituents (**39, 44**) and they were identified as *O*-methyl ellagic acid sulfate and *O*-dimethyl ellagic acid sulfate, respectively. Similar sulfate ellagic acid derivatives have been described from *Lagerstroemia speciosa* (Lythraceae) and *Euphorbia sororia* (Euphorbiaceae) (Bai et al., 2008; Zhang et al., 2008). The derivatives of ellagic acid were only detected from *C. adamantium* roots.

### Cytotoxic activity

**Figure 3:**
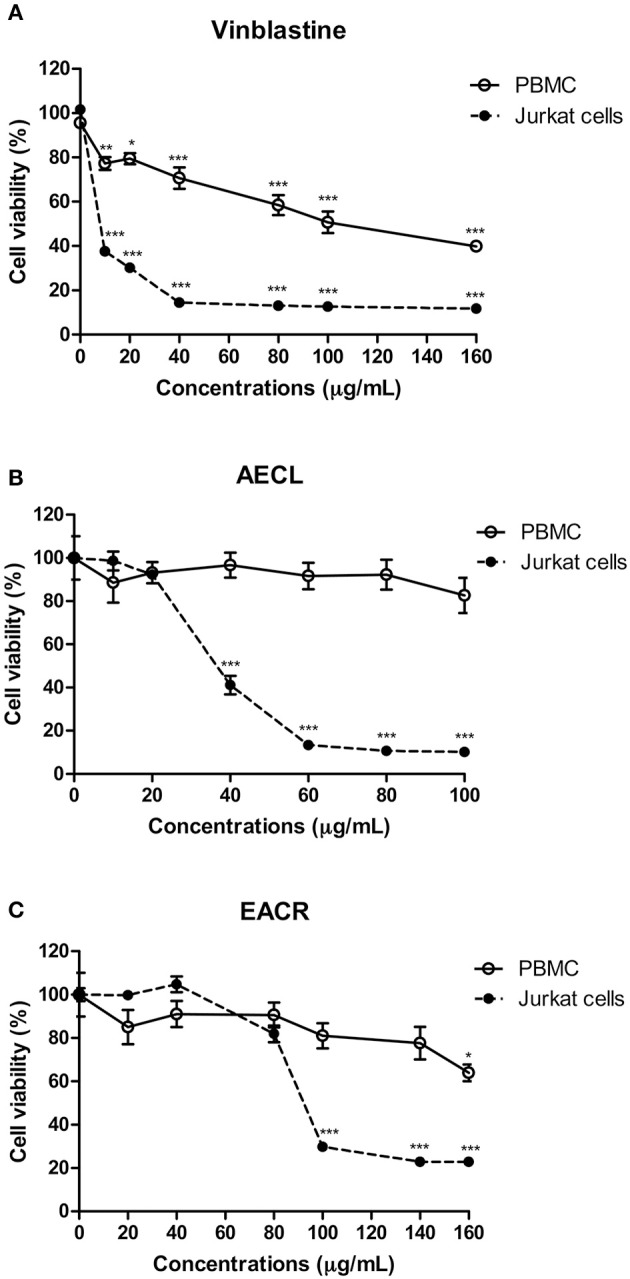
Viability of peripheral blood mononuclear cells (PBMC) and Jurkat cells treated with different concentrations of the vinblastine **(A)**, AECL **(B)**, and AECR **(C)**. ^*^p < 0.05, ^**^p < 0.01, and ^***^p < 0.001 compared with the control group.

Peripheral blood mononuclear cells and Jurkat cells were treated with vinblastine, AECL or AECR to assess cell viability. Vinblastine promoted cell death in Peripheral blood mononuclear cells (IC_50_ = 25.9 μg/mL) and Jurkat cells (IC_50_ = 9.5 μg/mL) (Figure 3A). The AECL did not change the viability of Peripheral blood mononuclear cells at the evaluated concentrations, however, promoted death in Jurkat cell (IC_50_ = 40 μg/mL) (Figure 3B). AECR showed low cytotoxicity against Peripheral blood mononuclear cells only at the highest concentration evaluated (160 μg/mL), but demonstrated concentration-dependent cytotoxicity against Jurkat cells (IC_50_ = 80 μg/mL) (Figure 3C).

### Cell death profile

**Figure 4:**
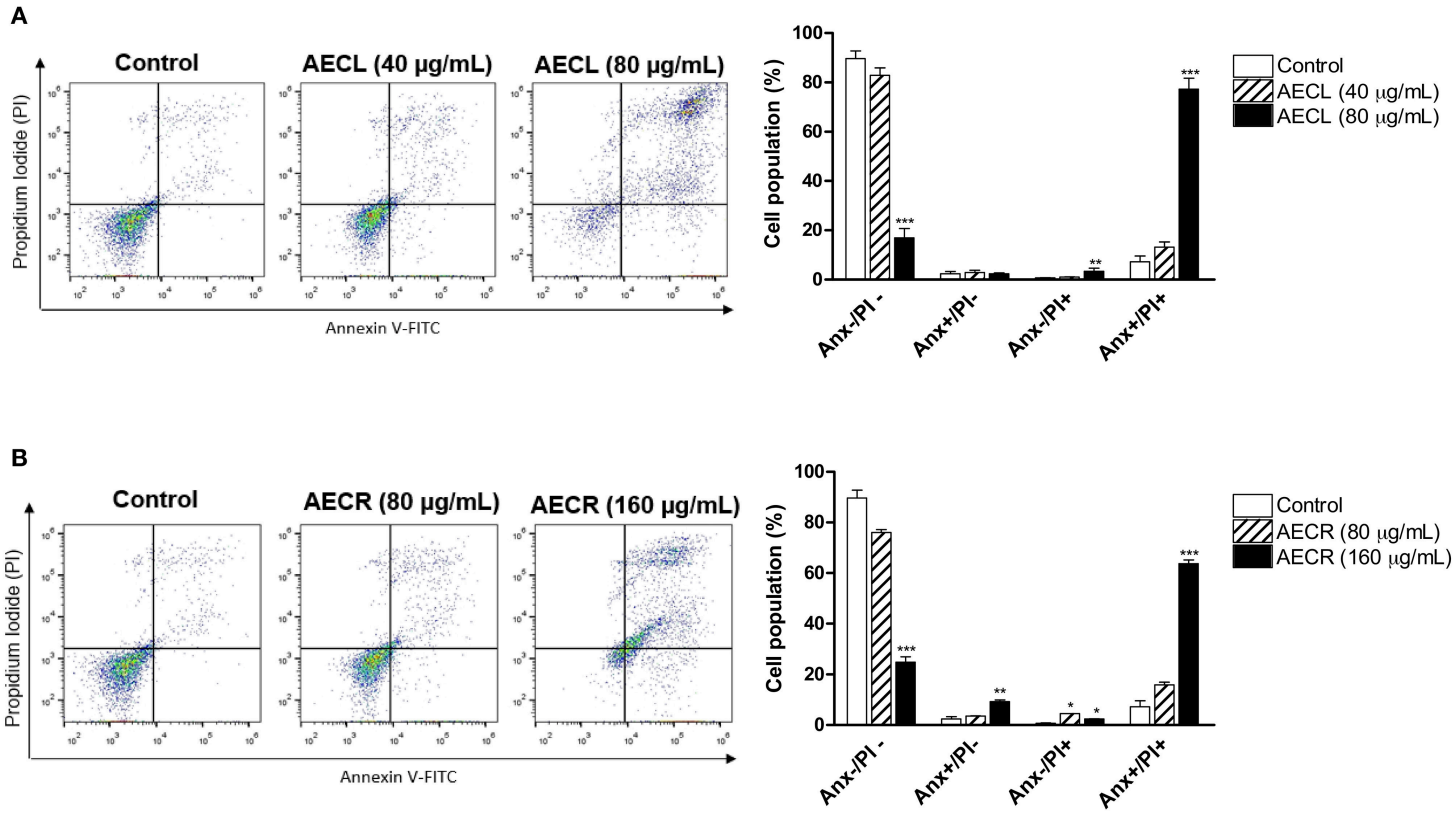
Cell death profile after treatment with AECL **(A)** and AECR **(B)**. Data obtained via flow cytometry of cells stained with annexin V-FITC/PI; Anx^–^/PI^–^: viable cells; Anx^+^/PI^–^: apoptotic cells; Anx^–^/PI^+^: necrotic cells, and Anx^+^/PI^+^: cells in late apoptosis. ^*^p < 0.05, ^**^p < 0.01, and ^***^p < 0.001 compared with the control group.

Both extracts resulted in double staining (annexin V^+^/PI^+^), indicating late apoptosis. This type of death was observed in 76.1 ± 3.2% of the cells treated with 80 μg/mL AECL (Figure 4A) and in 65.1 ± 1.4% of the cells treated with 160 μg/mL AECR (Figure 4B).

### Mitochondrial membrane potential (ΔΨmit)

**Figure 5:**
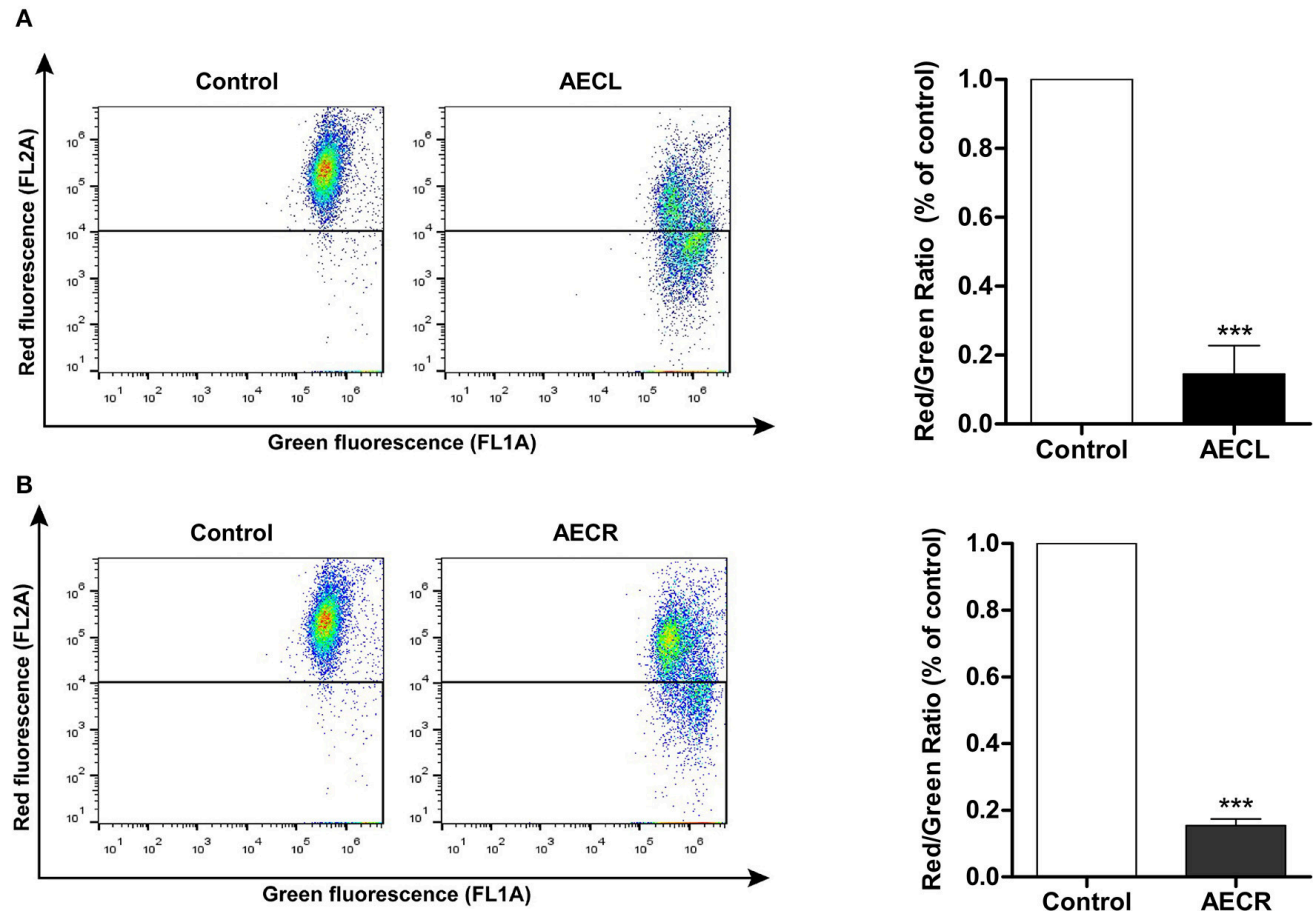
Mitochondrial membrane potential of Jurkat cells treated with AECL **(A)** and AECR **(B)**. ^***^p < 0.001 compared with the control group.

ΔΨmit was decreased in cells treated with AECL and AECR compared with untreated cells, as demonstrated by decreased red fluorescence and increased green fluorescence after a 24-h incubation. The rates of mitochondrial depolarization in cells treated with AECL and AECR were 85.4 ± 8.2 and 84.5 ± 2.0%, respectively (Figures 5A,B).

### Expression of caspases, Blc-2, and Bax

**Figure 6:**
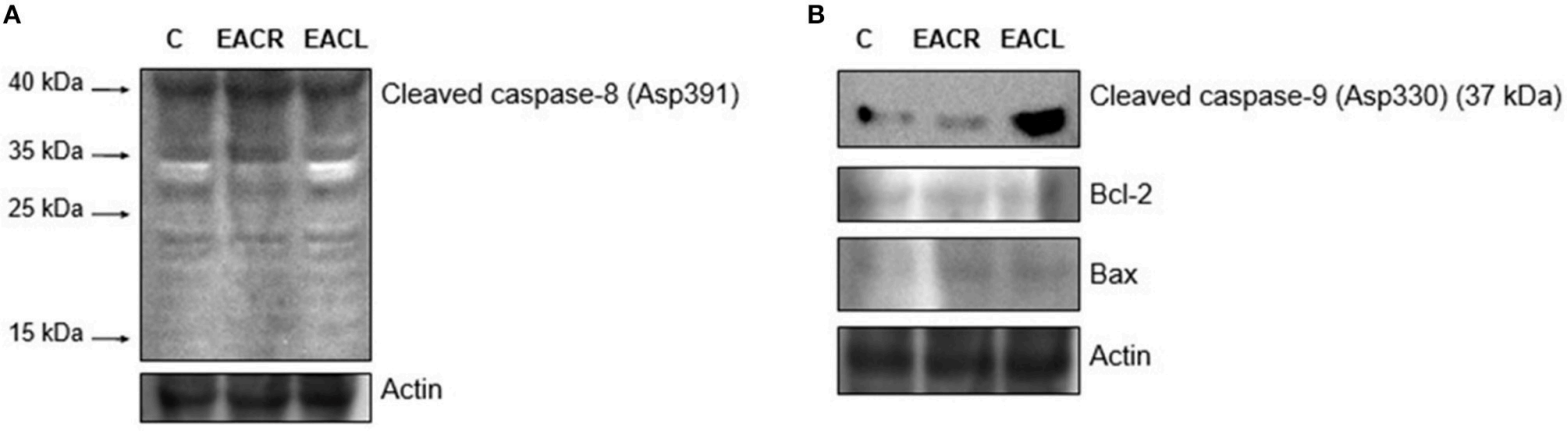
Western blot analysis. Jurkat Cells were treated with vehicle (control) or with the extracts for 24 h and were harvested for western blot analysis. The expression of **(A)** cleaved caspase-8 (Asp391) and **(B)** cleaved caspase-9 (Asp330) (37 kDa), Bax, Bcl-2 was determined by western blot. Equal loading was confirmed by reprobing blots for actin. One representative immunoblot of two independent experiments was presented.

In order to evaluate the role of the extrinsic and intrinsic pathways of apoptosis in cell death, protein expression of cleaved-caspases-8 and -9, Bcl-2, and Bax were investigated by western blot. As demonstrated in Figure 6A, the extrinsic pathway did not influence the mechanism of action of these extracts as evidenced by the absence of the characteristic fragments of cleaved caspase-8 (Asp391). In contrast, there was an increase in the cleaved form of caspase-9 (Asp330) in cellular extracts treated with both AECL and AECR, suggesting a role for the intrinsic pathway in mediating apoptosis. Moreover, this was followed by a slight increase in the expression of Bax, whereas no alteration was seen in Bcl-2 expression (Figure 6B).

**Figure 7:**
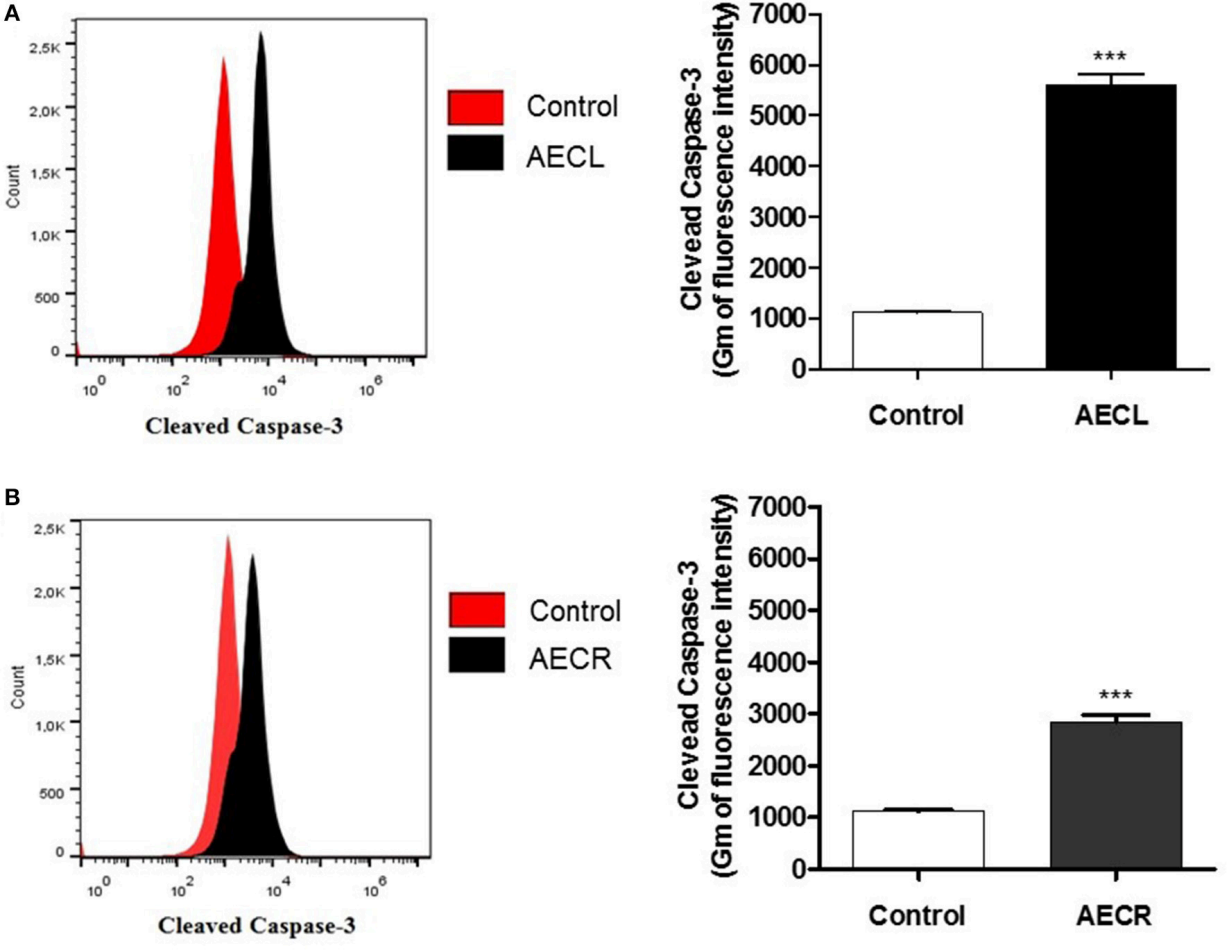
Caspase-3 activation in Jurkat cells treated with AECL **(A)** and AECR **(B)**. ^***^p < 0.001 compared with the control group.

A monoclonal anti-cleaved caspase-3 antibody was used to evaluate caspase-3 activation in cells incubated with AECL and AECR, and the cells were analyzed via flow cytometry. Both extracts resulted in the cleavage of pro-caspase 3, as indicated by a shift in fluorescence to the right (Figures 7A,B). Caspase-3 activation was 5.0-fold higher in cells treated with AECL (Figure 7A) and 2.5-fold higher in cells treated with AECR compared with the untreated control (Figure 7B).

### Pan-caspase inhibitor and intracellular calcium levels

**Figure 8:**
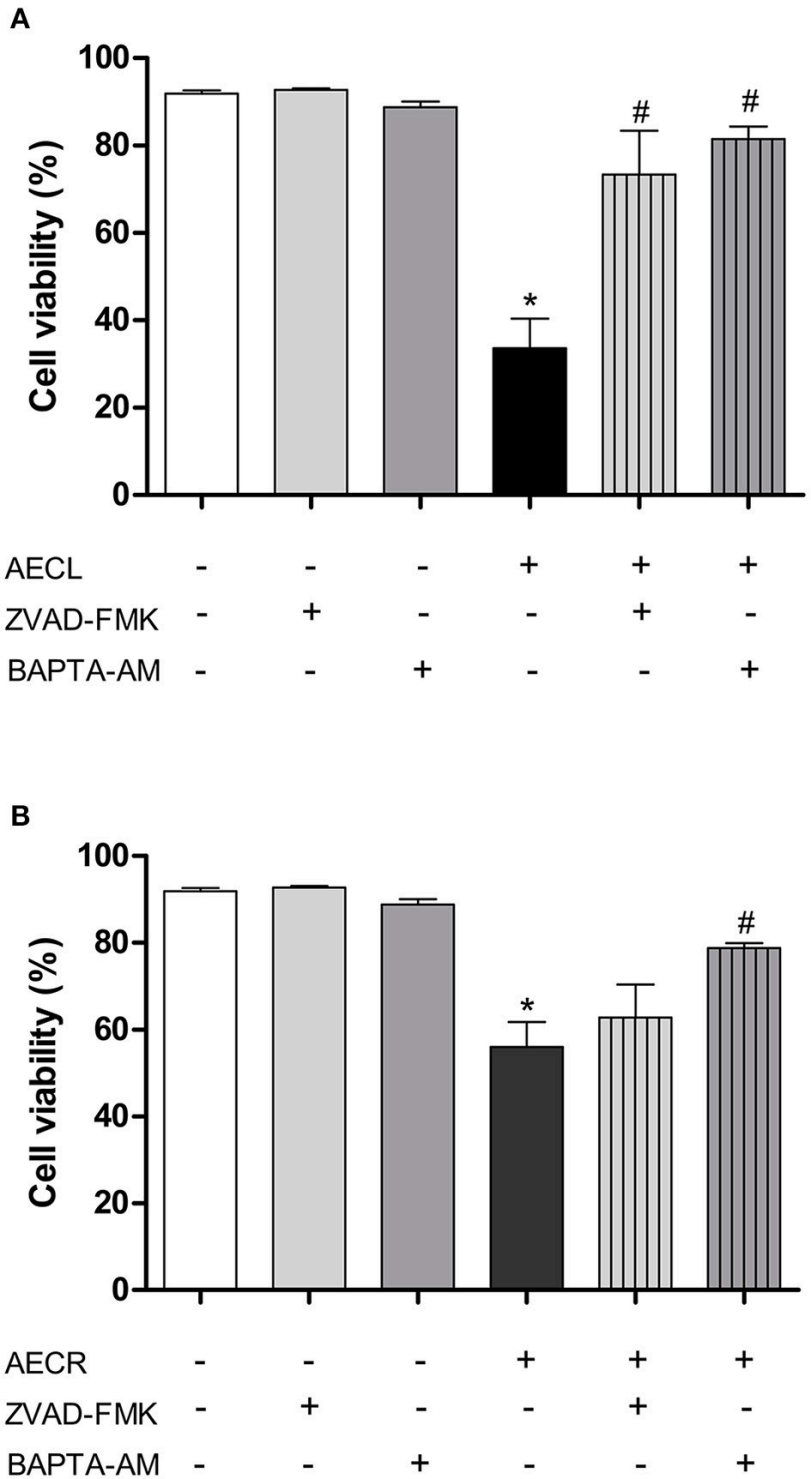
Effect of a pan-caspase inhibitor (Z-VAD-FMK) and an intracellular calcium chelator (BAPTA-AM) on cell death mediated by AECL **(A)** and AECR **(B)**. ^*^p < 0.05 compared with the control group. ^#^*p* < 0.05 compared with the AECL or AECR group.

Jurkat cells were pre-incubated with Z-VAD-FMK, a general caspase inhibitor, to assess whether caspases were involved in the cytotoxicity induced by the tested extracts. We observed that AECL-induced cytotoxicity decreased after treatment with the inhibitor (Figure 8A), indicating that caspases are actively involved in the cytotoxicity of this extract. However, pre-incubation with Z-VAD-FMK resulted in no difference in the viability of cells treated with AECR (Figure 8B).

Additionally, Jurkat cells were pre-incubated with BAPTA-AM, an intracellular Ca^2+^ chelator, to investigate the role of Ca^2+^ in the cytotoxicity induced by the extracts. Our results showed that AECL- and AECR-induced cytotoxicity was decreased after treatment with BAPTA-AM (Figures 8A,B), demonstrating the importance of calcium in the cytotoxic activity of the extracts in this cell line.

**Figure 9:**
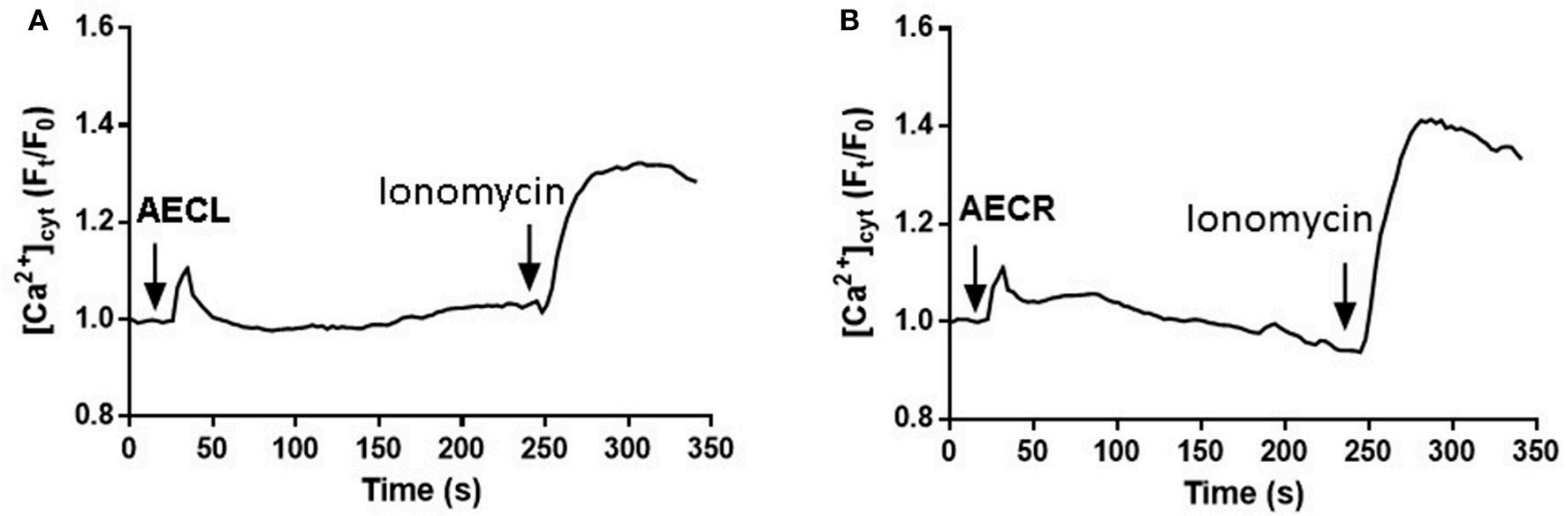
Increases in [Ca^2+^]_cyt_ induced by AECL **(A)** and AECR **(B)** in Jurkat cells. Cell were loaded with Fluo-4 Direct Calcium Assay Kit. The detection of fluorescence was performed in a FlexStation 3 microplate reader. Ionomycin was used as positive control to obtain the maximal fluorescence.

To corroborate the involvement of cytoplasmatic Ca^2+^ in cell death AECL and AECR- induced we performed a temporal evaluation of Ca^2+^ concentration. Both compounds were able to promoted increase of Ca^2+^ concentration in Jurkat cells (Figures 9A,B).

### Cell cycle distribution

**Figure 10:**
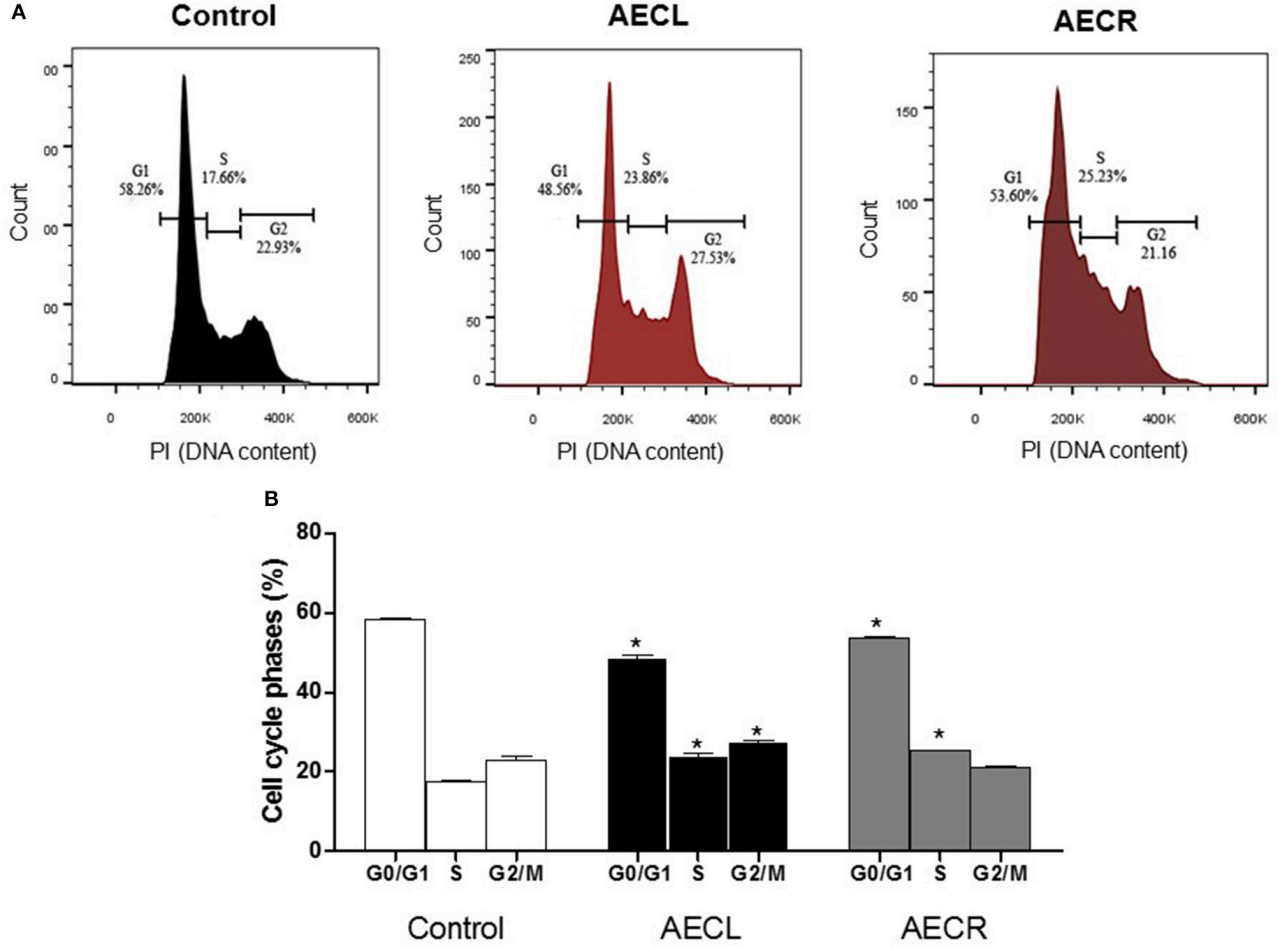
Cell cycle distribution after treatment with AECL **(A)** and AECR **(B)** for 24 h. ^*^p < 0.05 compared with the control group.

The effect of the extracts on the cell cycle distribution is shown in Figure 10A. The numbers of Jurkat cells in G1 phase were decreased by 16.6 ± 1.3 and 8.0 ± 0.5% after 24 h of treatment with AECL and AECR, respectively, compared with untreated cells. The number of cells in S phase after treatment with AECL and AECR increased to 23.86 and 25.23%, respectively, compared with untreated cells 17.66% (Figures 10A,B).

**Figure 11:**
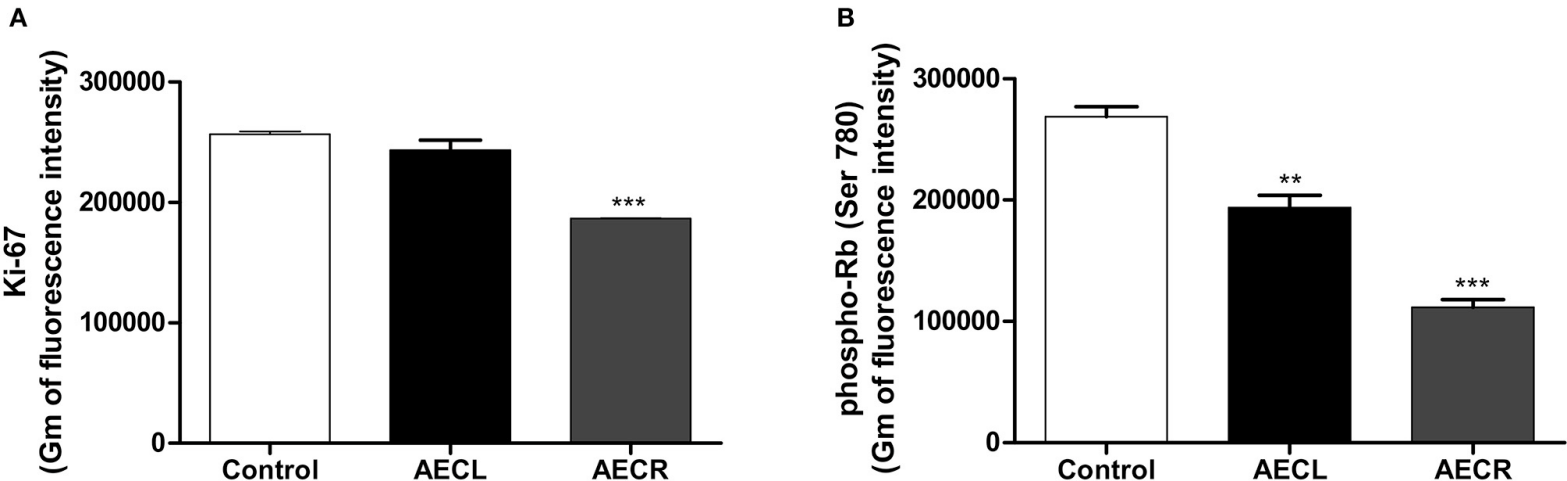
Quantification of Ki-67 **(A)** and p-Rb **(B)** in Jurkat cells treated with AECL and AECR. ^**^p < 0.01, and ^***^p < 0.001 compared with the control group.

The arrest of cell cycle was confirmed by the quantification of Ki-67 protein, a protein only expressed during the cell division cycle, and by quantification of p-Rb, a protein that prevent the progression of cell cycle when is not phosphorylated. Ki-67 was not altered by AECL, whereas AECR promoted a reduction of 27.3 ± 0.1% (Figure 11A). Furthermore, the cell cycle arrest was also confirmed by the reduction of p-RB levels (AECL 27.9 ± 3.7%; AECR 58.5 ± 2.5%) (Figure 11B).

## Discussion

The fruits of *C. adamantium* are widely consumed freshly, and to produce liqueurs, juice and sweets (Pavan et al., 2009). Several chemical and biological studies have been performed from the fruits (Pavan et al., 2009; Cardoso et al., 2010; Pascoal et al., 2014; Lescano et al., 2016; Souza et al., 2017). However, the studies from leaves and roots are still underexplored, representing a huge and important issue to comprehend their biological properties in this study and others.

The AECR and AECL showed huge differences from chemical constituents. AECR basically showed ellagic acid and its derivatives, (methoxylated, glycosylated, and sulfated), as well as organic acids in its composition. Differently, the AECL revealed mainly glycosylated flavonols, flavanones, flavan-3-ols, and condensed tannins. The main compounds identified from AECL were the flavonols myricetin *O*-pentoside (**23, 26, 29**), myricetin *O*-deoxyhexoside (**32**), quercetin *O*-pentoside (**38**), and myricetin *O*-(*O*-galloyl)-pentoside (**40**).

Brazilian biodiversity is rich in active compounds with high potential for development of new anticancer agents (Campos et al., 2016). Among the 174 anticancer drugs that have been commercially available in recent decades, ~10% are of natural origin, and 25% are derived from natural products (Newman and Cragg, 2016). These percentages underscore the need to the search for new sources of bioactive compounds for cancer therapy. In this study, the analysis of the cytotoxic potential of extracts of *C. adamantium* leaves and roots in leukemic Jurkat cells revealed that both extracts induced cell death; however, the leaf extract was more effective than the root extract.

These results suggest that most of the biologically active compounds found in this plant species are likely concentrated in the leaves, which showed chemical composition extremely different of roots and explain the differences at biological properties. Pascoal et al. (2014) showed that the compound cardamonin was predominantly found in extracts of the leaves and fruits of *C. adamantium*, and the higher antiproliferative activity associated with the leaves was correlated with the higher concentration of this phenolic compound.

In the present study, analysis of the cell death profile induced by the *C. adamantium* extracts demonstrated a predominance of late apoptotic death against Jurkat cells. This type of cell death, also known as post-apoptotic or secondary necrotic death (Patel et al., 2006), does not involve phagocytosis and is characterized by the activation of mechanisms similar to those of necrosis (Krysko et al., 2008). Late apoptotic death involves molecular and biochemical events typical of both apoptosis and necrosis. It is initiated by nuclear fragmentation, intensive chromatin condensation, and the release of activated caspase-3, which are typical events in apoptosis, leading to other activities that are characteristic of necrotic cell death, including cytoplasmic swelling, permeabilization of the cell membrane, and loss of membrane integrity (Silva, 2010).

Our results regarding the mechanism of action associated with the cytotoxicity of Jurkat cells indicated that both extracts promoted depolarization of the mitochondrial membrane. Dorn (2013) observed that apoptosis-mediated cell death is ATP dependent and requires energy to maintain mitochondrial respiration; thus, the opening of pores in the outer mitochondrial membrane is controlled by pro-apoptotic Bcl-2 family proteins, which bind to Bax and/or Bak molecules, in turn, contributing to the induction of apoptosis. In cells undergoing necrosis-induced death, the loss of ΔΨmit is ATP independent, and the opening of pores in the mitochondrial membrane is not controlled by other proteins, leading to osmotic swelling of the mitochondrial matrix and, consequently, expansion and rupture of the outer mitochondrial membrane (Proskuryakov et al., 2003; Dorn, 2013).

The cell death observed in the present study was likely mediated by the intrinsic apoptotic pathway, which involves the loss of ΔΨmit. The opening of pores in the mitochondrial membrane releases pro-apoptotic molecules, such as cytochrome C, from the intermembrane space of mitochondria to the cytosol, resulting in the formation of a complex known as the apoptosome, together with apoptotic protease activating factor-1 (APAF-1), procaspase 9, and ATP (Kim et al., 2003; Aguiar et al., 2012). In this process, procaspase 9 is cleaved and becomes caspase-9, which, in turn, cleaves procaspase-3 into caspase-3 (Proskuryakov et al., 2003; Li and Yuan, 2008). The last enzyme is partially or fully responsible for the proteolytic cleavage of critical proteins such as poly (ADP-ribose) polymerase (a DNA repair enzyme; Cohen, 1997) and the final stages of apoptosis (Sakulnarmrat et al., 2013).

To evaluate whether the extracts of the leaves and roots of *C. adamantium* promote the activation of caspases, protein expression of cleaved-caspases-8 and -9 were investigated by western blot and the monoclonal antibody anti-cleaved caspase-3 and a pan-caspase inhibitor were used. The activation of caspase-9 and -3 confirmed the involvement of the intrinsic pathway of apoposis in the cell death induced by both extracts. However, only the death of cells treated with AECL was reversed after incubation with the general caspase inhibitor.

Caspases are a family of aspartate-specific cysteine proteases present in the cell cytoplasm in an inactive form (known as procaspases) and are activated either by autoproteolysis induced by interaction with adapter proteins (death effector domains or caspase recruitment domains) or via cleavage by other proteases (Krysko et al., 2008). In addition to these mechanisms of action, calcium ions are involved in the death of Jurkat cells. Calcium is essential for the regulation of cell death in apoptosis by interacting with Bcl-2 family proteins such as Bax and Bid, which induce apoptosis via the mitochondrial pathway (Orrenius et al., 2015) and necrosis via intracellular Ca^2+^ overload, leading to loss of mitochondrial permeability (Lemasters et al., 1999; Festjens et al., 2006).

Calcium may activate Bax, which induces permeabilization of the mitochondrial membrane and release of mitochondrial pro-apoptotic factors, culminating in the activation of cell death effector caspases such as caspase-3 (Nutt et al., 2002), which is also activated by AECL and AECR.

The cytotoxic activity of *C. adamantium* extracts is associated with their chemical composition. Anticancer activities have been described for gallic and ellagic acid, both identified in AECL and AECR. The gallic acid triggers death in various cell lines via the intrinsic and extrinsic apoptotic pathways (Faried et al., 2007; Wang et al., 2014). Ellagic acid induces apoptosis in acute myeloid leukemia cells and is involved in the activation of caspase-3 (Hagiwara et al., 2010). In addition, this compound also induces changes on nuclear deoxyribonucleoside triphosphate (Saiko et al., 2015), and inhibits the proliferation of tumor cells by activating the TGF-β/Smad3 signaling pathway as well as proteins involved in cell proliferation and differentiation and by inducing cell cycle arrest (Zhang et al., 2014).

Cell cycle arrest is considered an effective strategy for eliminating cancer cells (Cho et al., 2011). The cell cycle is a complex process that involves interplay between several regulatory molecules, including cyclins and cyclin-dependent kinases (CDKs) (Zhu et al., 2015). The irregular metabolism of tumor cells is associated with failure of this process, resulting in uncontrolled cell proliferation (Zhang et al., 2015). The extracts evaluated in this study decreased the number of cells in G0/G1 phase and increased the number in S phase of the cell cycle.

Additionally, AECR reduces Ki-67 and p-Rb protein levels, involved in the mechanisms of cell cycle proliferation and arrest, respectively, while AECL decreases only p-Rb levels. Ki-67 is a nuclear protein detected during the cell cycle and it is a marker of cell proliferation (Torquato et al., 2017). The retinoblastoma (Rb) protein, when phosphorylated by the cyclin-CDK complexes, induces the expression of transcriptional factors such as E2F, promoting the advancement of the cycle (Hamilton and Infante, 2016). Therefore, compounds that reduces proliferation and/or leads to death of cancer cells through different mechanisms are of paramount importance, particularly when they are effective in leukemic cell lines that are resistant to certain types of cell death.

From AECL, several glycosylated flavonols of quercetin and myricetin aglycones were detected, which could also be related to the activity, since the inhibition on the growth of human promyelocytic leukemia cells were reported for quercetin 3-O-β-D-glucopyranoside (Kim et al., 2000). For the non-glycosylated flavonols quercetin, kaempferol and myricetin, the proteasome-inhibition, and apoptosis-inducing activities have been reported in human tumor cells (Chen et al., 2005), as well as the structure-activity relationships of flavonoids for cytotoxicity on human leukemia cells (Plochmann et al., 2007). From this study, the structural features for higher activity was determined, such as the carbon-carbon double bond on C ring, the carbonyl on C-4, the hydroxylation on B ring and other. Some of these features can be found in the flavonoids from *C. adamantium* extracts, but there are not wide studies from its glycosylated derivatives and also for the ellagic acid derivatives, mainly for leukemic cells.

Other studies also demonstrate the antileukemic potential of plant extracts, including mechanisms similar to those presented by extracts of *C. adamantium*, such as the reduction of mitochondrial membrane potential (Casagrande et al., 2014; Santos et al., 2016), increase Bax protein expression, activation of caspases-9 and -3, and cell cycle arrest (Asmaa et al., 2014; Fan et al., 2016). Furthermore, the AECL showed similar citotoxicity to the crude extract of *Pereskia sacharosa* leaves (Asmaa et al., 2014), and higher than those obtained by extracts from leaves of *P. sacharosa* (Asmaa et al., 2014), *Jacaranda decurrens* (Casagrande et al., 2014), and *Hancornia speciosa* (Santos et al., 2016).

In conclusion, this study revealed for the first time that aqueous extracts of the leaves and roots of *C. adamantium* exert antileukemic activity via late apoptosis by decreasing the mitochondrial membrane potential, increasing the activation of caspase-9 and 3, and intracellular calcium levels. Hence, these extracts show therapeutic potential for use in the prevention and treatment of diseases associated the proliferation of tumor cell. Besides, the extensive chemical identification of the constituents from AECL and AECR were determined, demonstrating huge chemical differences between the extracts that can explain the biological properties.

## Author contributions

JC, PE, HT, WV, GJ, DS, CC, KdP, EdS, and Ed contributed to the literature database search, data collection, data extraction, data analysis, and writing of the manuscript. JC, DS, CC, Kd, EP, and Ed performed data analysis and rationalization of the results. All the authors read and approved the final version of the manuscript.

### Conflict of interest statement

The authors declare that the research was conducted in the absence of any commercial or financial relationships that could be construed as a potential conflict of interest.
